# A comparative study for tension-reducing effect of Type I and Type II keystone perforator island flap in the human back

**DOI:** 10.1038/s41598-021-96272-0

**Published:** 2021-08-17

**Authors:** Chi Sun Yoon, Yu Taek Kong, Soo Yeon Lim, Junekyu Kim, Hyun Woo Shin, Kyu Nam Kim

**Affiliations:** 1 Department of Plastic and Reconstructive Surgery, Wonkwang University Hospital, Wonkwang University School of Medicine, Iksan, Korea; 2 Department of Plastic and Reconstructive Surgery, Kangbuk Samsung Hospital, Sungkyunkwan University School of Medicine, Seoul, Korea; 3 Department of Plastic and Reconstructive Surgery, Konyang University Hospital, University of Konyang College of Medicine, Myunggok Medical Research Center, Daejeon, Korea

**Keywords:** Anatomy, Medical research

## Abstract

The keystone perforator island flap (KPIF) is popular in reconstructive surgery. However, despite its versatility, its biomechanical effectiveness is unclear. We present our experience of KPIF reconstruction in the human back and evaluate the tension-reducing effect of the KPIF. Between September 2019 and August 2020, 17 patients (51.82 ± 14.72 years) underwent KPIF reconstruction for back defects. In all cases, we measured wound tension at the defect and donor sites before and after KPIF reconstruction using a tensiometer. All defects occurred after complete excision of complicated epidermoid cysts and debridement of surrounding tissues. The defects were successfully covered with Type IIA KPIFs. All flaps survived, and there were no significant postoperative complications. The mean “tension change at the defect after Type I KPIF” and “tension change at the defect after Type II KPIF” were − 2.97 ± 0.22 N and − 5.59 ± 0.41 N, respectively, (*P* < 0.001). The mean “rate of tension change at the defect after Type I KPIF” and “rate of tension change at the defect after Type II KPIF” were − 36.54 ± 1.89% and − 67.98 ± 1.63%, respectively, (*P* < 0.001). Our findings confirm the stepwise tension-reducing effect of KPIF and clarify the biomechanics of this flap.

## Introduction

The keystone perforator island flap (KPIF), a curvilinear-shaped trapezoidal flap devised by Behan in 2003, has been a popular reconstructive flap and a good alternative to other flaps for the past 20 years^1–3^. Fundamentally, the KPIF is an island-shaped advancement flap based on the presence of random multi-perforators arising from fasciocutaneous or musculocutaneous perforators, which are often called central hot spots of perforators^1–9^. Thus, the center of the KPIF should be located near these hot spots, and the orientation of its longitudinal axis should correspond with the long axis of the defect to maximally capture the dominant perforators with linking vessels, thereby securing stable and reliable flap perfusion^3,7^. Behan considered that the vascularity of the KPIF was suprafascial, and intraracial network circulation from the perforator axis was randomly located; however, it must be located within the dermatomal precincts because the blood supply must run along with a nerve supply^1,5^. In addition to these vascular support basics, KPIF has a unique biomechanical property − the recruitment of tissue laxity^3,8,10^. As the keystone in Roman arches locks other stones in place, the KPIF locks into the defect through the fusion of two opposing V–Y flaps^1,3,7^. Behan’s original concept of the KPIF flap is that either end of the V–Y advancement flap facilitates the recruitment of surrounding tissues and the redistribution of skin tension perpendicular to the direction of flap advancement, which confers a tension-reducing effect on the KPIF^3,8–10^. In addition, Behan classified the KPIF into the following four subtypes: type I (skin incision only), type II (A, division of the deep fascia along the outer curvilinear line; B, division of the deep fascia and skin graft to the secondary defect), type III (double-opposing keystone flaps), and type IV (keystone flap with undermining of up to 50% of the flap subfascially)^1^. Currently, KPIF-based reconstruction is widely used both as a primary and an alternative method in reconstructive surgery, and various studies have demonstrated the application of KPIF for covering diverse defects of the body^2–6^. The characteristics of the KPIF, including its simple defect-adaptive design, easy reproducibility, and stability due to its sturdy vascular supply, may increase the popularity of KPIF-based reconstruction^1–8^. After Behan’s original paper, which reported successful outcomes in a series of 300 patients^1^, using the KPIF has been commonly associated with the significant benefit of minimizing the necessity of skin grafting or microsurgery in patients who are unwilling to undergo these procedures^1–3,6,8,11^.

Despite the versatility and usefulness of the KPIF, there have been differences of opinion regarding the biomechanical effectiveness and rationale of using this flap. Specifically, it has been argued whether KPIF is truly effective in reducing wound tension and promoting wound closure^10,12–16^. Some authors claimed that wound closure using the KPIF flap did not reduce wound tension and, thus, questioned the ability of the KPIF skin paddle to expand, based on the results of their in vitro (cadaveric) study^13,16^. They mentioned that, unlike the KPIF, the V–Y flap had biomechanical benefits^13,16^. Thus, these authors concluded that the KPIF technique is not mechanically superior to direct closure and has no “closablility” for unclosable wounds^16^. Contrarily, our study group previously validated the tension-reducing effect of KPIF in the back area of the human body by measuring wound tension before and after KPIF reconstruction using a tensiometer^8^. Since Behan devised the KPIF technique, a number of surgeons have clinically applied the KPIF to cover defects in various regions of the body, with successful outcomes. In this regard, we support the practical utility of the KPIF. In our previous study, we substantiated the tension-reducing effect of the Type II KPIF in reconstructive procedures involving the human back. Based on the results of our previous study, we planned the present study, which compares the tension-reducing effect of Type I KPIF with that of Type II KPIF.

In the present study, we present a retrospective review of our KPIF reconstruction in the back area and evaluate the tension-reducing effect of the KPIF technique with (Type II KPIF) and without (Type I KPIF) the division of the deep fascia using tensiometer measurements. The main purpose of this study was to clarify the stepwise tension-reducing effect of the KPIF technique, which can contribute to the understanding of the biomechanical benefits of the KPIF.

## Materials and methods

This study was approved by the ethical review board of Konyang University Hospital (approval number: 2020-08-017). All research procedures in this study were performed in accordance with the ethical guidelines of the 1975 Declaration of Helsinki. Written informed consent was obtained from all patients. All patients of our study provided consent to publish the information and images in an online open-access publication.

In the present study, we retrospectively reviewed patients who underwent KPIF coverage of back defects between September 2019 and August 2020. We included patients who received only Type II KPIF (division of the deep fascia and primary closure of the donor site) and excluded those who received other types of KPIF, such as Type IIB, Type III, and Type IV KPIF. In Behan’s original classification, Type IIA KPIF was defined as a division of the deep fascia along the outer curvilinear line and primary closure of the donor site, but we regarded Type II KPIF as a case of a division of the deep fascia along the whole circumference of the KPIF and primary closure of the donor site in this study. All KPIF reconstructions were performed by the senior author of this study. We collected and reviewed patient data from medical records and clinical photographs, including data regarding the cause and location of defects, defect sizes, flap sizes, flap survival, postoperative complications, and the follow-up duration of each patient.

### Intraoperative tensiometer measurement

**Figure 1:**
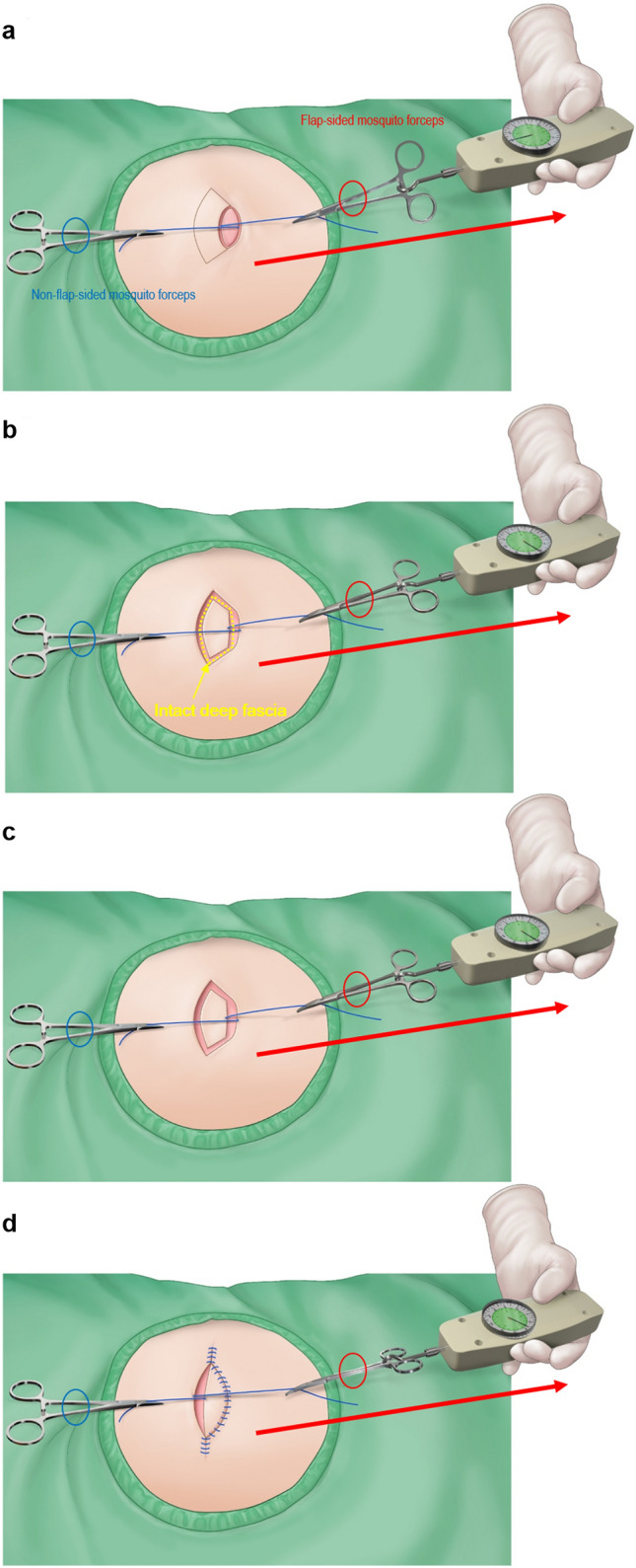
Schematic illustration of the procedure for intraoperative tensiometer measurement. A surgeon passes 3–0 nylon sutures through both wound edges at the most concave point and attaches mosquito forceps to each suture. The surgeon then connects the tensiometer to the flap-sided forceps and gently pulls it toward the non-flap-sided forceps until the two wound edges are as approximate as possible. At this time, the non-flap-sided forceps are not pulled but are kept in place as the reference point for the direction of the flap-sided forceps. The surgeon performed three consecutive measurements for each parameter in this way and calculated the average value. In all these procedures, the tensiometer should be sterilized for intraoperative use. The red-colored oval represents the flap-sided mosquito forceps, and the blue oval represents the non-flap-sided mosquito forceps. (**a**) Measurement of “pre-flap tension at the defect.” (**b**) Measurement of “Type I keystone perforator island flap (KPIF) tension at the defect.” The yellow dotted line represents the intact deep fascia before division. (**c**) Measurement of “Type II KPIF tension at the defect.” (**d**) Measurement of “post-flap tension at the donor.” (Figures (**a**), (**c**), and (**d**) were reprinted from Yoon et al.^8^, with permission from Springer Nature).

Intraoperative tension measurements at the defect and donor sites before and after KPIF reconstruction were performed with a tensiometer (Analog Force Gauge, Wenzhou Tripod Instrument Manufacturing Co., Ltd, Zhejiang, China), as previously described^8^. Figure 1 shows a schematic illustration of the procedure we employed for intraoperative tension measurement. We defined the following parameters of tensiometric measurement and recorded the values for each patient: (1) “Pre-flap tension at the defect” (Fig. 1a), which signifies tension measurement across the widest point of the defect before KPIF reconstruction; (2) “Type I KPIF tension at the defect” (Fig. 1b), which refers to tension measurement across the widest point of the defect after conducting skin incision and releasing the thick dermis and subcutaneous tissues, without division of the deep fascia; (3) “Type II KPIF tension at the defect” (Fig. 1c), which signifies tension measurement across the widest point of the defect after the division of deep fascia; and (4) “Post-flap tension at the donor” (Fig. 1d), which refers to tension measurement across the widest point of the donor site after the closure of the defect-side flap and V–Y closure of either end of KPIF. For example, to measure A, we passed 3–0 nylon sutures through both wound edges at the most concave point and attached mosquito forceps to each suture. Subsequently, we connected the tensiometer to the flap-side forceps and gently pulled it toward the non-flap-side forceps until the two wound edges were as approximated as possible. At this time, the non-flap-side forceps were not pulled but were kept in place as a reference point for the direction of the flap-side forceps. We performed three consecutive measurements for each parameter (A, B, C, and D) in this way and calculated the average value.

### Surgical techniques

In case of lesions with inflammation of surrounding tissues, such as cellulitis, empirical antibiotic treatment was administered until the infection subsided for 1–2 weeks. Then, sufficient wound preparation and stabilization were achieved, and we performed the KPIF reconstruction.

After complete excision and debridement of the lesion in the back area, the final defect was reached, and A was measured and recorded (Fig. 1a). At the time of KPIF design, we considered two factors: the surrounding tissue laxity and the relaxed skin tension lines (RSTLs)^2,3,8,9^. We designed the KPIF to fit either the upper or lower side of the defect, whichever had more tissue laxity, and created the long axis of the KPIF parallel to the RSTLs of the back area, which are located transversely^8^. The width of the KPIF was designed to be slightly larger than that of the defect based on the two abovenamed factors^2,3,8,9^. Skin incisions were made along the designed KPIF, and dissection was performed from the thick dermis layer to the subcutaneous tissue layer in the first step. At this time, we measured and recorded B (Fig. 1b). The next step was the division of the deep fascia along the whole circumference of the KPIF, and C was measured and recorded (Fig. 1c). After creating the island-shaped flap structure, the margin of the KPIF was minimally undermined to obtain further flap movement while preserving the central hot spots of vascular perforators^2,3,8,9^. The procedure of insetting the flap, following fastidious bleeding control, was performed as previously described^8^. First, the defect site of the KPIF was sutured. At this time, it was very important to suture the flap on the defect layer-by-layer to avoid dead space formation. Second, both ends of the KPIF were sutured in a V–Y closure fashion. Immediately after the closure, D was measured and recorded (Fig. 1d). Lastly, the donor site of the KPIF was sutured. Mild compressive dressing with a foam material was applied at the end of the operation.

### Statistical analysis

#### Software and basic statistics

We used R language version 3.3.3 (R Foundation for Statistical Computing, Vienna, Austria) and STATA version 15 (StataCorp, College Station, TX) software for all statistical analyses. Continuous and categorical variables are expressed as mean ± standard deviation (SD) and sample number and percentage (N (%)), respectively.

#### Comparison of the mean difference of paired variables

The difference between paired variables such as between A, B, and C or between A and D is expressed as mean ± SE (standard error). We used the Wilcoxon signed-rank test to evaluate whether the mean difference between the paired variables was zero. The significance level was set at a *p-value* of < 0.05.

## Results

Seventeen patients (10 male and seven female patients), with an average age of 51.82 ± 14.72 (range, 8–84) years, were included in this study. Table 1 shows a summary of the patients’ characteristics and their clinical data.

**Table 1:** Patient characteristics and clinical data.

Case	Sex/age (yrs)	Diagnosis	Location	Defect size (cm)	Flap size (cm)	Flap survival	Complication	Follow-up periods (months)
1	F/41	Ruptured epidermoid cyst	Upper right back	5 × 5	6 × 12	Fully survived	None	11
2	F/43	Ruptured epidermoid cyst	Upper right back	3.5 × 3.5	4 × 8	Fully survived	None	10
3	M/72	Ruptured epidermoid cyst	Upper right back	3 × 4	4 × 9	Fully survived	None	9
4	M/71	Ruptured epidermoid cyst	Upper middle back	4.5 × 5	5 × 12	Fully survived	None	8
5	M/42	Ruptured epidermoid cyst with surrounding cellulitis	Upper left back	6 × 8	8 × 18	Fully survived	None	6
6	M/22	Ruptured epidermoid cyst	Upper middle back	3 × 3.5	3.5 × 7	Fully survived	Wound dehiscence → debridement and delayed closure	6
7	M/70	Ruptured epidermoid cyst with surrounding cellulitis	Lower left back	3 × 5	4 × 10	Fully survived	None	5
8	M/55	Ruptured epidermoid cyst with surrounding cellulitis	Middle left back	6 × 8	7.5 × 16	Fully survived	None	5
9	M/24	Ruptured epidermoid cyst	Upper left back	2 × 3	2.5 × 6	Fully survived	None	5
10	F/59	Ruptured epidermoid cyst	Upper middle back	2 × 3	2.5 × 6.5	Fully survived	None	4
11	F/44	Ruptured epidermoid cyst with surrounding cellulitis	Middle right back	4 × 6.5	5 × 12	Fully survived	None	4
12	M/63	Ruptured epidermoid cyst with surrounding cellulitis	Upper middle back	4 × 6	5 × 10	Fully survived	None	4
13	M/58	Ruptured epidermoid cyst	Upper left back	2.5 × 3.5	3 × 7	Fully survived	None	7
14	F/54	Ruptured epidermoid cyst with surrounding cellulitis	Lower right back	3.5 × 5	3.5 × 10	Fully survived	None	6
15	F/55	Ruptured epidermoid cyst	Upper right back	2 × 3.5	3 × 7	Fully survived	None	6
16	M/61	Ruptured epidermoid cyst	Middle right back	3 × 4	4 × 7.5	Fully survived	Marginal maceration → spontaneous healing with conservative dressing	5
17	F/ 47	Ruptured epidermoid cyst	Lower left back	3 × 3.5	3.5 × 7.5	Fully survived	None	6

*M* male, *F* female.

This study analyzed 17 cases of ruptured epidermoid cysts, including 6 cases of concomitant cellulitis of surrounding tissues. All defects occurred after the complete excision of primary cystic lesions, followed by the debridement and adhesiolysis of unhealthy surrounding tissues. All defects, ranging from 2 × 3 cm to 6 × 8 cm, were successfully covered with Type IIA KPIFs, ranging from 2.5 × 6 to 8 × 18 cm. Complete flap survival was observed without flap-related complications, such as venous congestion and arterial insufficiency, in all cases. Small-sized wound dehiscence occurred in one patient, and this was managed by wound debridement, followed by delayed closure. Marginal maceration occurred in another patient, but the maceration was treated conservatively. All patients were satisfied with the final outcome of the procedure at an average of 6.29 ± 2.08 (range, 4–11) months after the procedure.

**Figure 2:**
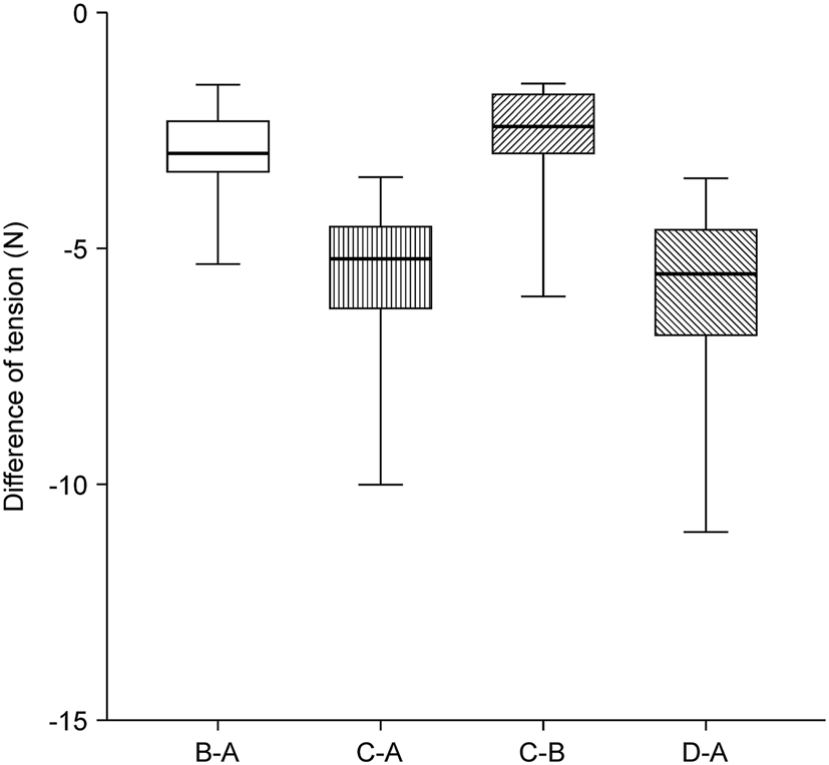
Comparison of mean differences between paired variables of tension change tensiometer data. (a) pre-flap tension at the defect; (b) Type I keystone perforator island flap (KPIF) tension at the defect; (c) Type II KPIF tension at the defect; (d) post-flap tension at the donor.

**Figure 3:**
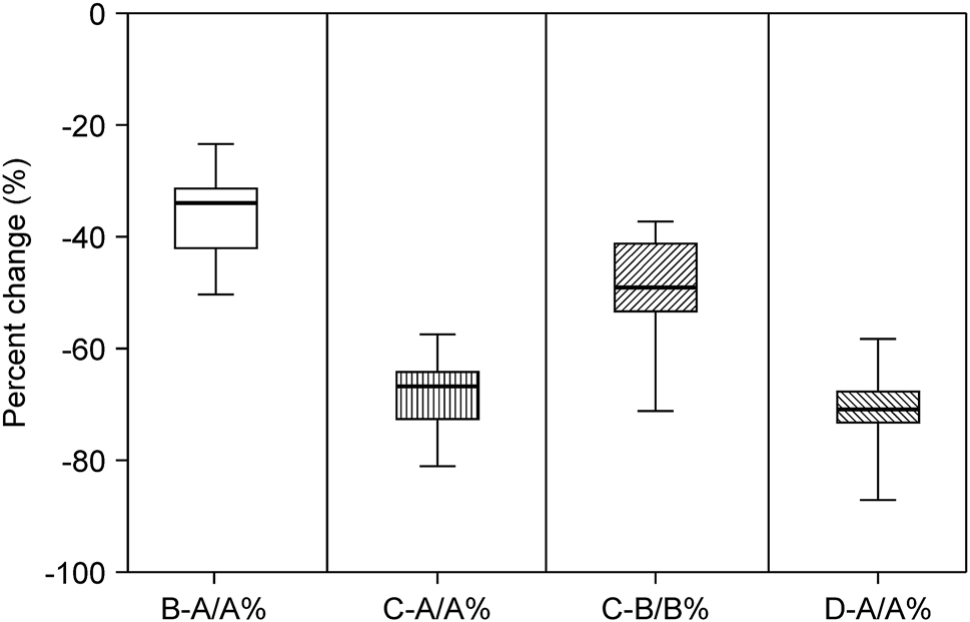
Comparison of mean differences between paired variables of the rate of tension change tensiometer data. (a) pre-flap tension at the defect; (b) Type I keystone perforator island flap (KPIF) tension at the defect; (c) Type II KPIF tension at the defect; (d) post-flap tension at the donor.

**Figure 4:**
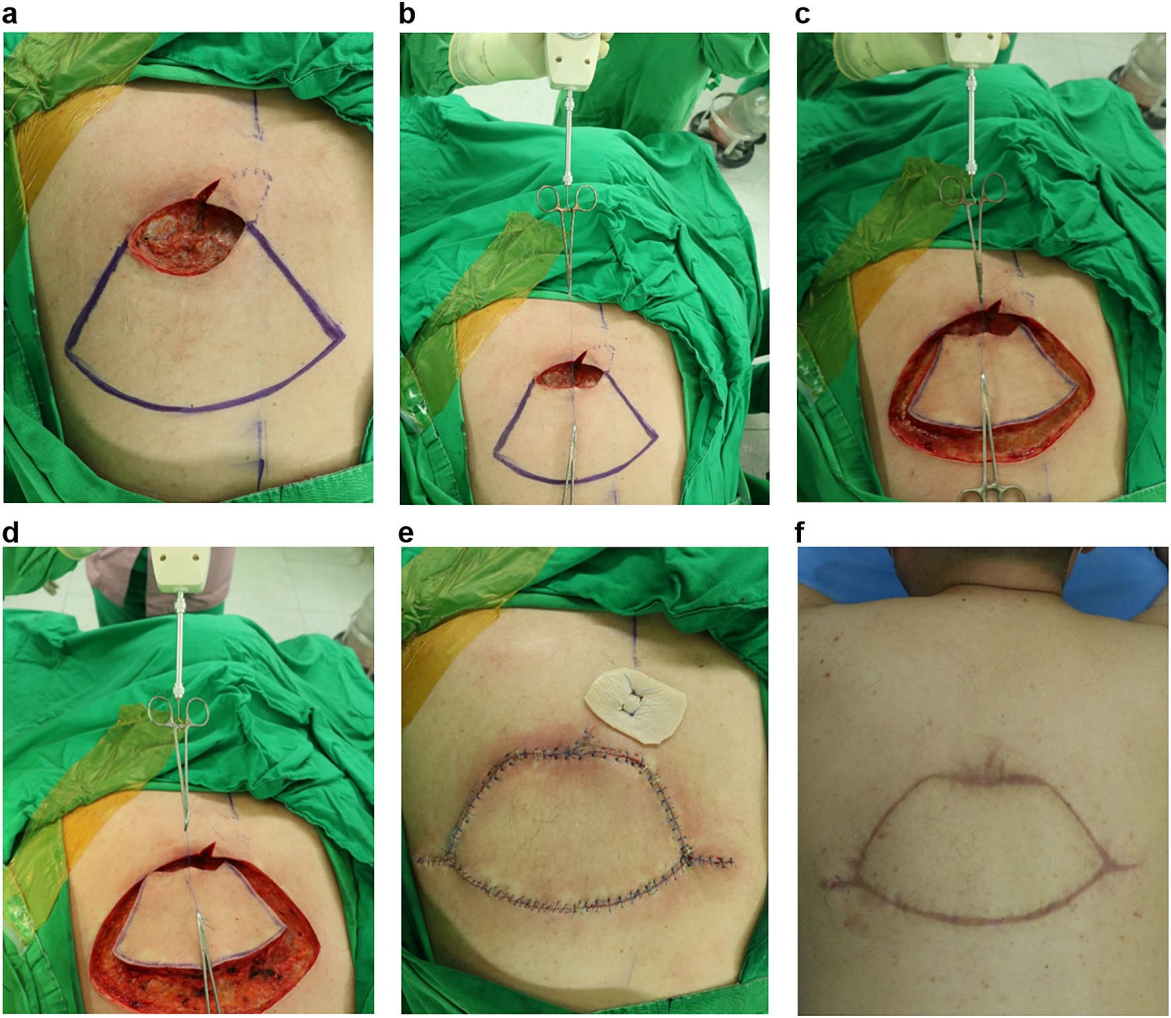
Keystone perforator island flap (KPIF) coverage for a defect that resulted from complete excision of a ruptured epidermoid cyst with cellulitis in a 42-year-old man. (**a**) After complete excision and debridement of the lesion in the upper left back, the final defect size is 6 × 8 cm. We designed an 8 × 18 cm-sized KPIF in the lower side of the defect. (**b**) Before performing skin incisions, the “pre-flap tension at the defect” is measured and recorded. (**c**) After the release of the thick dermis and subcutaneous tissues, with no division of the deep fascia, “Type I KPIF tension at the defect” is measured and recorded. (**d**) After the division of the deep fascia, “Type II KPIF tension at the defect” is measured and recorded. (**e**) Immediate postoperative clinical photograph. (**f**) Six-month follow-up clinical photograph.

**Figure 5:**
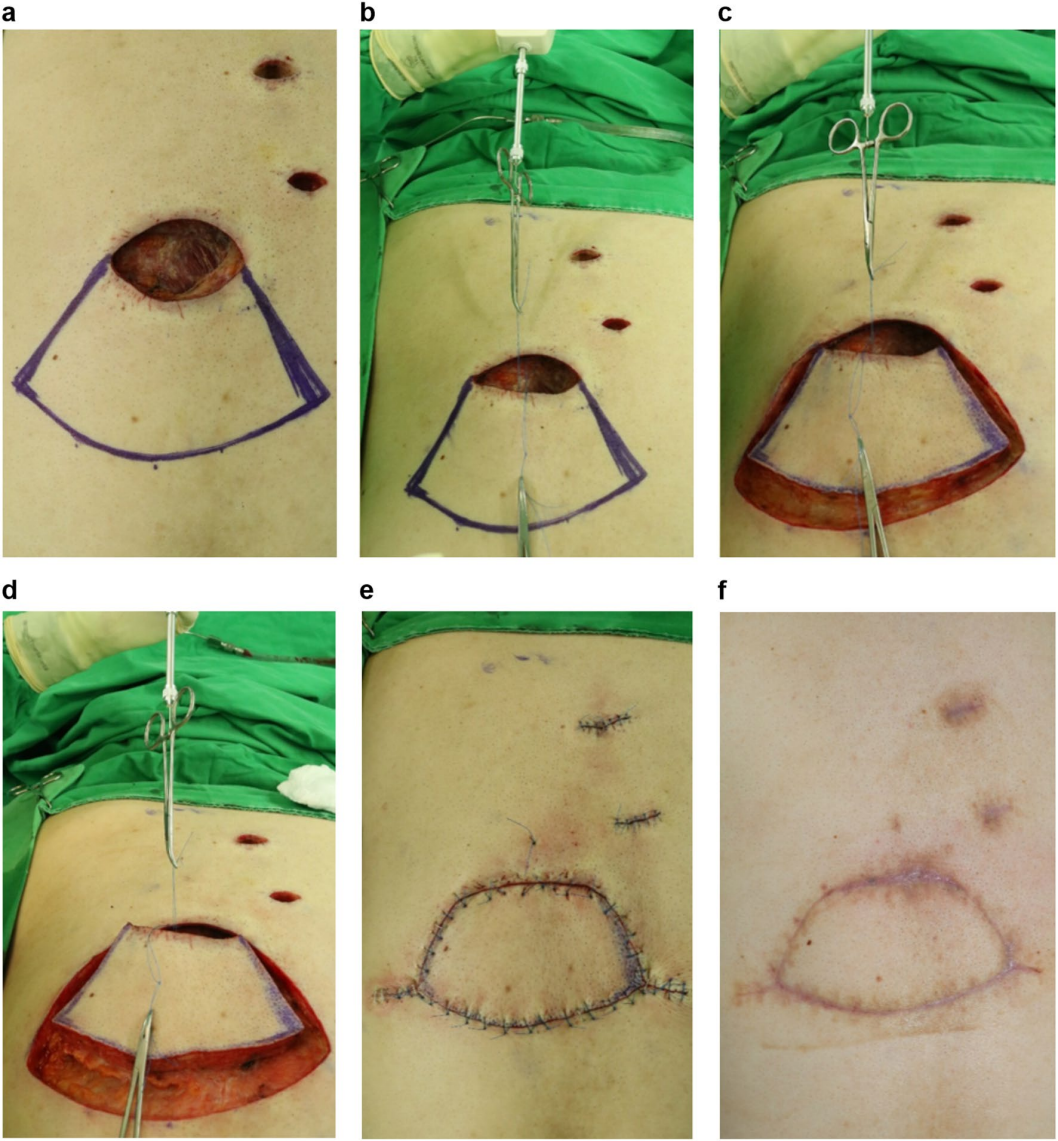
Keystone perforator island flap (KPIF) coverage for a defect that resulted from complete excision of a ruptured epidermoid cyst with cellulitis in a 55-year-old man. (**a**) After complete excision and debridement of the lesion in the middle left back, the final defect size is 6 × 8 cm, and a 7.5 × 16 cm-sized KPIF is designed in the lower side of the defect. (**b**) Before performing skin incisions, “pre-flap tension at the defect” is measured and recorded. (**c**) After the release of the thick dermis and subcutaneous tissues, without the division of the deep fascia, “Type I KPIF tension at the defect” is measured and recorded. (**d**) After the division of the deep fascia, “Type II KPIF tension at the defect” is measured and recorded. (**e**) Immediate postoperative clinical photograph. (**f**) Six-month follow-up clinical photograph.

Tables 2 and 3 show the tensiometer data of each patient and a summary of the variables of tension measurement, respectively. The mean values of A, B, C, and D were 8.26 ± 0.59 N, 5.29 ± 0.46 N, 2.67 ± 0.23 N, and 2.35 ± 0.16 N, respectively. The mean “tension-change at the defect after performing Type I KPIF” (B-A) and the mean “tension-change at the defect after performing Type II KPIF” (C-A) were − 2.97 ± 0.22 N and − 5.59 ± 0.41 N, respectively, (*P* < 0.001). The mean “rate of tension-change at the defect after performing Type I KPIF” (B-A/A%) and the mean “rate of tension-change at the defect after performing Type II KPIF” (C-A/A%) were − 36.54 ± 1.89% and − 67.98 ± 1.63%, respectively, (*P* < 0.001). Thus, both the Type I and Type II KPIF tension levels at the defect sites showed a significant diminution in comparison with the level of pre-flap tension at the defect. The mean “tension change between Type I and Type II KPIF” (C-B) and the mean “rate of tension change between Type I and Type II KPIF” (C-B/B%) were − 2.62 ± 0.28 N and − 49.31 ± 2.40%, respectively, (*P* < 0.001). Thus, tension levels after the division of the deep fascia were found to have significantly decreased compared to the pre-division levels. The mean “tension change at the donor” (D-A) and the mean “rate of tension change at the donor” (D-A/A%) were − 5.91 ± 0.48 N and − 70.99 ± 1.55%, respectively, (*P* < 0.001). Accordingly, the post-flap tension levels at the donor sites showed a significant decrease compared with the level of pre-flap tension at the defect site. Figures 2 and 3 show a comparison of mean differences between paired variables of tension change and the rate of tension change, respectively. We have provided clinical photographs of representative cases (Figs. 4 and 5) to clarify our experience of KPIF reconstruction of back defects.

**Table 2:** Tensiometer data.

Case	A. Pre-flap tension at the defect (N)	B. Type I KPIF tension at the defect (N)	C. Type II KPIF tension at the defect (N)	D. **Post**-flap tension at the donor (N)	Tension-change at the defect after Type I KPIF (B-A, N)	Tension-change at the defect after Type II KPIF (C-A, N)	Tension-change between Type I and Type II KPIF (C-B, N)	Tension-change at the donor (D-A, N)	Rate of tension-change at the defect after Type I KPIF (B-A/A%)	Rate of tension-change at the defect after Type II KPIF (C-A/A%)	Rate of tension-change between Type I and Type II KPIF (C-B/B%)	Rate of tension-change at the donor (D-A/A%)
1	11	5.7	3.5	3	− 5.3	− 7.5	− 2.2	− 8	− 48.18	− 68.18	− 38.59	− 72.73
2	7	4.2	2.6	2	− 2.8	− 4.4	− 1.6	− 5	− 40	− 62.86	− 38.09	− 71.43
3	6	3	1.5	1.5	− 3	− 4.5	− 1.5	− 4.5	− 50	− 75	− 50	− 75
4	7.5	4	2.5	2.5	− 3.5	− 5	− 1.5	− 5	− 46.67	− 66.67	− 37.5	− 66.67
5	9.5	7	4	3	− 2.5	− 5.5	− 3	− 6.5	− 26.32	− 57.89	− 42.86	− 68.42
6	8	4.5	1.5	1	− 3.5	− 6.5	− 3	− 7	− 43.75	− 81.25	− 66.67	− 87.5
7	12	8	4	3.5	− 4	− 8	− 4	− 8.5	− 33.33	− 66.67	− 50	− 70.83
8	14	10	4	3	− 4	− 10	− 6	− 11	− 28.57	− 71.43	− 60	− 78.57
9	5.5	3.5	1.8	2	− 2	− 3.7	− 1.7	− 3.5	− 36.36	− 67.27	− 48.57	− 63.64
10	5	3.5	1	1.5	− 1.5	− 4	− 2.5	− 3.5	− 30	− 80	− 71.43	− 70
11	9	6	3	2.5	− 3	− 6	− 3	− 6.5	− 33.33	− 66.67	− 50	− 72.22
12	9.5	6.5	3.2	2.5	− 3	− 6.3	− 3.3	− 7	− 31.58	− 66.32	− 50.77	− 73.68
13	8.5	6.5	3.3	2.7	− 2	− 5.2	− 3.2	− 5.8	− 23.53	− 61.18	− 49.23	− 68.24
14	7.6	4.5	2.5	2.5	− 3.1	− 5.1	− 2	− 5.1	− 40.79	− 67.11	− 44.44	− 67.11
15	6	3.5	1.5	1.5	− 2.5	− 4.5	− 2	− 4.5	− 41.67	− 75	− 57.14	− 75
16	8.3	5.5	3	2.7	− 2.8	− 5.3	− 2.5	− 5.6	− 33.73	− 63.86	− 45.45	− 67.47
17	6	4	2.5	2.5	− 2	− 3.5	− 1.5	− 3.5	− 33.33	− 58.33	− 37.5	− 58.33

*N* Newton, *KPIF* keystone perforator island flap.

**Table 3:** Summary of tensiometer data.

Variables	Mean ± SE	*P*-value
Mean value of the pre-flap tension at the defect (A, N)	8.26 ± 0.59	NA
Mean value of the Type I KPIF tension at the defect (B, N)	5.29 ± 0.46	NA
Mean value of the Type II KPIF tension at the defect (C, N)	2.67 ± 0.23	NA
Mean value of the post-flap tension at the donor (D, N)	2.35 ± 0.16	NA
Mean value of tension-change at the defect after Type I KPIF (B-A, N)	− 2.97 ± 0.22	*P* < 0.001
Mean value of tension-change at the defect after Type II KPIF (C-A, N)	− 5.59 ± 0.41	*P* < 0.001
Mean value of tension-change between Type I and Type II KPIF (C-B, N)	− 2.62 ± 0.28	*P* < 0.001
Mean value of tension-change at the donor (D-A, N)	− 5.91 ± 0.48	*P* < 0.001
Mean value of the rate of tension-change at the defect after Type I KPIF (B-A/A%)	− 36.54 ± 1.89	*P* < 0.001
Mean value of the rate of tension-change at the defect after Type II KPIF (C-A/A%)	− 67.98 ± 1.63	*P* < 0.001
Mean value of the rate of tension-change between Type I and Type II KPIF (C-B/B%)	− 49.31 ± 2.40	*P* < 0.001
Mean value of the rate of tension-change at the donor (D-A/A%)	− 70.99 ± 1.55	*P* < 0.001

All variables, difference between paired variables, and rate of change are expressed as mean ± SE; *P* values were obtained using Wilcoxon signed-rank test.

*N* Newton, *KPIF* keystone perforator island flap, *SE* standard error.

## Discussion

The present study, which is an extension of our previous study, evaluated the stepwise tension-reducing effect of the KPIF technique between patients who received Type I KPIF and those who received Type II KPIF for the reconstruction of defects in the back area. We demonstrated a single surgeon’s experience with KPIF reconstruction and investigated the tension-reducing effect of KPIF by comparing Type I and Type II KPIFs in this study. The overall outcomes of the present study suggest that both Type I and Type II KPIFs have tension-reducing effects, and that Type II KPIF has greater tension-reducing ability than Type I KPIF in the human back area. The significant mean values of B-A/A% and C-A/A% prove the tension-reducing effect of Type I and II KPIFs. The significant mean values of C-B/B% prove the superior tension-reducing ability of Type II KPIF compared with that of Type I KPIF.

As noted earlier, the study by Donovan et al. compared the wound tension change and wound closure ability of three methods, including V–Y flap reconstruction, KPIF-based reconstruction, and primary closure, in fresh-frozen cadavers^16^. The results of their study showed that V–Y flaps enabled the closure of “unclosable” defects and produced a significant drop in wound tension across the primary defect. However, KPIFs did not close unclosable defects and failed to achieve tension reduction^16^. Contrary to the study by Donovan et al., other researchers who advocate the usefulness of KPIF or are unsure about it have claimed the contrary, based on the following: the skin is a complex organ with diverse characteristics including non-linear, anisotropic, and viscoelastic properties, and it is difficult to interpret the results of in vitro (cadaveric) testing of skin tension because of established differences between in vivo and in vitro settings^10,12^. Overall, there seems to be no clear consensus on whether KPIF reconstruction can reduce wound tension. However, our senior author has performed collaborative KPIF reconstructions including the face, trunk, back, and extremities since 2015^2,3,6,8,9,17^, and we fully agree that KPIF reconstruction is useful and has advantages, such as a defect-adaptive design, stability (drawn from sturdy vascular support), and easy reproducibility^1–8^. These advantages of the KPIF-based reconstruction are summarized by the acronym, P.A.C.E – P, the KPIF is a relatively pain-free technique; A, the KPIF provides esthetically like-for-like tissues; C, complications are relatively rare in the KPIF reconstruction from a vascular viewpoint; E, the KPIF has good economics in terms of time-saving^18^. In 2019, we published a paper on the coverage of back defects using KPIFs, and it was confirmed through tensiometer measurements that wound tension decreased when Type II KPIFs were applied to close defects in the back^8^. As mentioned earlier, the results of our previous research provide the practical basis for the present study. We consider that some differences exist between our research and that of Donovan et al., as follows. First, unlike the study by Donovan et al., we performed an in vivo study, which reflects the real characteristics of the human skin and tissues^8^. Second, Donovan et al.’s study was based on cadaveric upper and lower extremities^16^, while we performed KPIF reconstruction on the human back^8^. The ratio of tissue components across the body varies depending on the body part. In other words, the tissue component of an area may affect the viscoelastic property of that region, which can lead to different results in measurements of change in wound tension. However, we consider that the skin of the back is thick and both the subcutaneous tissue and the fascia layer are clearly formed in the back area^8^; therefore, differences in mechanics and kinetics between tissue layers could reflect in KPIF reconstruction in the back. Third, the insetting procedure of KPIF in our studies followed Behan’s original sequence. First, we sutured the defect, then we sutured both ends with V–Y closure and sutured the donor side^8^. However, Donovan et al. performed both V–Y closure and donor-side suturing first, followed by defect-side suturing^16^. Generally, it is a principle of the local flap technique that the defect is covered through various flap movements, such as transposition, rotation, and advancement, followed by the closure of the donor site. Thus, the order of flap insetting and closure applied by Donovan et al. is contrary to this reconstructive principle, which may have introduced bias and error in their outcomes.

The movement of KPIF is fundamentally achieved by the step-wise release of tissue layers, including the skin (including the dermis), subcutaneous layer (including the superficial fascia), and deep fascia, during flap dissection^3,8^. While constructing the island flap in KPIF back reconstruction, operating surgeons can easily note a gradual slack in the force-holding tissues surrounding the flap, particularly owing to a sequential release of the thick dermis of the back, followed by the release of the superficial fascia and the thoracolumbar fascia of the back^8^. Our previous study demonstrated wound tension reduction in KPIF reconstruction in an in vivo experiment in which a significant decrease in the pre-flap tension level at the defect site, compared with the post-flap tension levels at both the defect and donor sites in the human back, was noted^8^. This finding indicates that the Type II KPIF reconstruction, which involves division of the deep fascia, has a tension-reducing effect^3,8^. A previous study also confirmed a drop in tension by the deep fascia release in both the traditional KPIF and the percutaneous fasciotomy through a fresh cadaver study^19^.

Fascia means deep fascia to many surgeons, but it can anatomically be divided into superficial and deep fascia^20^. The superficial fascia is typically considered as the layer of areolar connective tissue immediately beneath the skin, whereas the deep fascia is the sturdy and thick connective tissue that is continuous with the superficial fascia^20^. The subcutaneous fat layer is divided into superficial and deep fat layers by the superficial fascia, and the deep fascia is located below the subcutaneous tissue layer and above the muscle layer^8^. According to Behan's original classification, Type I KPIF involves only skin incision without the division of the deep fascia^1^. We consider that the movement of Type I KPIF can be attributed to both the release of the skin layer, including the thick dermis, and the subcutaneous layer, including the superficial fascia of the back^8^. We measured B, which is gauged after the release of the subcutaneous layer and before the division of the deep fascia, to evaluate the effectiveness of Type I KPIF in reducing tension. In our study, the mean values of B-A and B-A/A% were − 2.97 ± 0.22 N and − 36.54 ± 1.89%, respectively, indicating that the division of the superficial fascia could result in a significant reduction of wound tension in the KPIF. To the best of our knowledge, no previous study has demonstrated the tension-reducing effect of Type I KPIF. Using tensiometric measurements, the present study confirmed that the Type I KPIF has a tension-reducing effect in reconstructions of the human back area. However, both the mean values of C-B and C-B/B% were significantly decreased in the present study. Thus, we consider that Type II KPIF has a greater tension-reducing effect than Type I KPIF, and the division of the deep fascia does not only reduce more wound tension than the division of the superficial fascia but also plays an important role in the dynamics and mechanics of the KPIF. Although Type II KPIF has a greater tension reducing-effect than Type I KPIF based on the results of the present study, it cannot be said that the effectiveness of Type I KPIF as a reconstructive modality is inferior to that of Type II KPIF. In case of defects possible to be covered by using Type I KPIF, there is no need to use Type II KPIF because Type I KPIF is more advantageous than Type II KPIF in terms of donor site morbidity. In case of defects difficult to be covered by using only Type I KPIF, we consider that Type II KPIF is useful in terms of effective coverage with less wound tension. These considerations are in line with those of the previous study, which mentioned that should more advancement be required for wound closure, the Type I KPIF must be turned into a Type II KPIF or Type III KPIF^21^. Additionally, both the mean C-A/A% (− 67.98 ± 1.63%) and D-A/A% (− 70.99 ± 1.55%) were significantly decreased in the present study, which is similar to the outcomes of our previous study in which the mean values of C-A/A% and D-A/A% were − 69.48 ± 1.7% and − 71.16 ± 1.33, respectively^8^. These outcomes further confirm our previous indication that Type II KPIF is effective in reducing wound tension in the back area^8^.

Although we successfully achieved KPIF reconstruction and evaluated the tension-reducing effect of KPIF reconstruction in the back region, the present study had several limitations. First, the KPIF reconstructions both in our past and present studies involved the back area alone. Therefore, our outcomes do not represent the dynamics and effect of KPIF in other parts of the human body. Second, we evaluated only Types I and II KPIF techniques in our study. It seems important to evaluate the tension-reducing effect of KPIFs of other types and modifications, such as Type IV KPIF, omega variation KPIF, and Sydney Melanoma Unit modification KPIF. Third, the present study was a retrospective clinical review with a non-randomized and non-blinded design, a comparatively small sample size, and no control group. Therefore, selection and confounding biases may have affected the outcomes. Fourth, we used an analog tensiometer for wound tension measurements in the present study. This might lead to some inaccuracy and errors in measurements compared with the use of the latest digital tension measuring instruments. Despite these limitations, it seems notable that our study is the first clinical proof of the tension-reducing ability of Types I and II KPIFs in reconstructive procedures of the human body. Future well-designed prospective studies with larger sample sizes and use of the latest digital tensiometer are required to confirm the consistency and validity of our study outcomes.

## Conclusions

Consistent with the outcomes of our previous study, we verified the tension-reducing effect of KPIF and clarified the stepwise tension reduction effect of KPIF-based reconstruction in achieving successful KPIF reconstruction of the back. We believe that the outcomes and findings of the present study introduce a valuable understanding of the tension-reducing effect of the KPIF, which can be a step forward to investigating the scientific knowledge of the KPIF reconstruction, especially regarding its dynamics and biomechanics. We intend to conduct large-scale studies with a prospective design in the future to confirm the consistency and validity of our outcomes and examine the effect of KPIF reconstruction in other areas of the body, to clarify the tension-reducing effect of KPIF in the human body.
